# Importance of deepening integration of crime and conservation sciences

**DOI:** 10.1111/cobi.13710

**Published:** 2021-02-18

**Authors:** Meredith L. Gore, Abigail Bennett

**Affiliations:** ^1^ Department of Geographical Sciences University of Maryland College Park MD 20742 U.S.A.; ^2^ Department of Fisheries and Wildlife Michigan State University East Lansing MI 48824 U.S.A.

**Keywords:** common pool resources, community‐based management, crime science, environmental criminology, fisheries, governance, Mexico, sea cucumber, ciencia criminológica, criminología ambiental, gobernanza, manejo basado en la comunidad, México, pepino de mar, pesquerías

## Abstract

Conservation crime is a globally distributed societal problem. Conservation crime science, an emerging interdisciplinary field, has the potential to help address this problem. However, its utility depends on serious reflection on the transposition of crime science approaches to conservation contexts, which may differ in meaningful ways from traditional crime contexts. We considered the breadth of crime science approaches being used in conservation as well as the depth of crime science integration in conservation. We used the case of sea cucumber (*Holothuria floridana*, *Isostichopus badionotus*) trafficking in Mexico as an example of why the interdisciplinarity of crime and conservation sciences should be deepened and how integration can help ideate new solutions. We first conducted a review of literature to capture the range of interdisciplinarity applications. We identified 6 crime science approaches being applied to the conservation contexts of illegal, unreported, and unregulated fishing; wildlife and plant crime; and illegal logging. We then compared this knowledge base to the case of illegal sea cucumber fishing in Mexico. We identified 5 challenges in the application of these approaches to conservation contexts: the relative diffusion of harms and victims in conservation crimes; scalar mismatches in crime, authority, and the conservation issue itself; interactions between legal and illegal networks; communities and their authority to define and control crime; and the role of natural science in the rule of law. Considering these 5 factors may enhance the depth of interdisciplinarity between crime and conservation sciences. Nurturing interdisciplinary crime and conservation science will expand innovation and help accelerate successful risk management programs and other policy agendas.

## Abstract 2

*Article impact statement*: Sea cucumber trafficking in Mexico exemplifies the value and challenges of deepening interdisciplinarity of crime and conservation sciences.

## Abstract (es)

La Importancia de Profundizar la Integración de las Ciencias Criminológicas y de la Conservación Gore & Bennett

**Resumen:**

El crimen en conservación es un problema social distribuido globalmente. La ciencia criminológica de la conservación, un campo interdisciplinario emergente, tiene el potencial para contribuir a atender este problema. Sin embargo, su utilidad depende de la reflexión seria sobre la transposición de los métodos de la ciencia criminológica en el contexto de la conservación, que puede diferir de forma significativa de los de contextos tradicionales del crimen. Consideramos la gama de métodos de las ciencias criminológicas que están siendo utilizadas en conservación, así como la profundidad de la integración de la ciencia criminológica en la conservación. Utilizamos el caso del tráfico de pepino de mar (*Holothuria floridana, Isostichopus badionotus)* en México como un ejemplo de porqué la interdisciplinaridad de las ciencias criminológicas y de conservación debería profundizarse y de cómo puede la integración ayudar a idear nuevas soluciones. Primero hicimos una revisión de literatura para capturar el rango de aplicaciones interdisciplinarias. Identificamos que 6 métodos de ciencia criminológica fueron aplicados en contextos de conservación de pesca ilegal, no registrada y no regulada, crimen con fauna y plantas silvestres y tala ilegal. Posteriormente comparamos esta base de conocimiento al caso de la pesca ilegal de pepino de mar en México. Identificamos 5 retos en la aplicación de estos métodos en contextos de conservación: la difusión relativa de daños y víctimas en crímenes de conservación; desajustes escalares en el crimen, la autoridad y el tema de la conservación misma; interacciones entre redes legales e ilegales; comunidades y su autoridad para definir y controlar el crimen; y el papel de las ciencias naturales en el estado de derecho. Considerar estos 5 factores puede aumentar la profundidad de la interdisciplinaridad entre las ciencias criminológicas y de la conservación. Fomentar la ciencia criminológica y de conservación interdisciplinaria ampliará la innovación y ayudará a acelerar los programas exitosos de gestión de riesgos y otras agendas políticas.

## Introduction

Conservation crime—deviant human use of natural resources—can manifest across any context in the world with diverse negative effects. Conservation crimes involve illegal logging, mining, fishing, and wildlife poaching and trafficking. These activities can threaten endangered species, undermine resource‐dependent livelihoods, contribute to species declines that are both a cause and a consequence of social conflict, and converge with other serious crimes, such as drug trafficking, narco‐deforestation, and money laundering. Reducing risk to people and ecosystems from conservation crime is a cross‐cutting policy priority (e.g., Sustainable Development Goal 15: Life on Land; Goal 14: Life below Water; Goal 16: Peace, Justice and Strong Institutions). Public perceptions of risk to biodiversity from conservation crime have reached unprecedentedly high levels (Collins 2019). Scientific recognition of the scope, scale, and impact of crime in conservation is growing (e.g., Solomon et al. 2015; Gore et al. 2019).

Understandably, interdisciplinary research on conservation crime is yielding new programs and policies (e.g., Viollaz & Gore 2019). Some argue strongly that disciplinary research is not sufficient to study the social–ecological and human–environment interactions associated with phenomena such as conservation crime (Liu et al. 2007). Traditionally, the science of why and how people commit crimes has had little connection to the science of human behavior as it relates to natural resources, and vice versa, yet there is a clear opportunity to overlay the 2 disciplinary approaches, such as in contexts where natural resource governance faces persistent enforcement challenges (e.g., from lack of legitimacy, militarization, corruption, lack of resources). With increasing attention on implementing interdisciplinary approaches to understand and ideate solutions for conservation crime (e.g., United Nations Office on Drugs and Crime's Education for Justice Tertiary Education Online Module on Wildlife and Forest Crime), now is an appropriate time to reflect on the links among biodiversity conservation, natural resources governance disciplines, and crime science. These areas are opportunities for innovation and integrative discovery.

Interdisciplinary approaches inherently challenge traditional planning and management assumptions, power structures, and knowledge bases; they are important for policy making, governance, and management of natural resources (Liu et al. 2007). Understanding the state of knowledge about the interdisciplinary science of conservation crime (e.g., Gibbs et al. 2010; Gore 2017; Lynch & Pires 2019) is vital if it is to be nurtured. To this end, we first sought to identify the breadth of crime science approaches currently being deployed in conservation. Second, we examined the depth of disciplinary integration between crime and conservation sciences. We used the case of sea cucumber trafficking in Mexico as an exemplar for both objectives as to why interdisciplinarity of crime and conservation sciences should be deepened. Based on the sea cucumber case and key strands of thinking from natural resource governance literatures, we devised 5 avenues for deepening interdisciplinarity.

## Criminogenic Thinking for Conservation

Crime is a behavior that violates the law. Scientific studies of crime explore the causes, consequences, prevention, and nature of criminal behavior at different levels (e.g., individual, group, organizational). The conservation community overwhelmingly focuses on criminal (i.e., rule of law) and normative (i.e., rule in use) dimensions of human–environment relationships. Crime can be a social and moral construct; harm may derive from behavior that is not a violation of the rule of law. Some scholars argue crime is best prevented through the criminal justice system by arrest, prosecution, and treatment. The implications of this view are that crime management is the primary responsibility of police authorities and the state and partnerships between law enforcement and communities are secondary (e.g., Christie 2000).

In contrast, natural resource governance disciplines (e.g., scholarship on commons governance, political ecology, policy studies) problematize the role of the state in natural resource management, emphasize the importance of resource user involvement in defining and addressing conservation problems, and explore collaborative approaches (e.g., comanagement) to sustainable governance (Ostrom 1990; Robbins 2011). In worst‐case scenarios, law enforcement represents the deployment of state monopolies on lawful violence in the name of fighting conservation crime, which brings unjust violence against resource user communities (e.g., Peluso 1993; Peluso & Watts 2001) and disenfranchises already disadvantaged and vulnerable people while advancing the ends, norms, and values of the powerful (e.g., nongovernmental organizations, parastatals, foundations, donors) (Robbins 2011). In best‐case scenarios, state regulatory regimes are engaged in monitoring and enforcement of compliance, supporting community‐based natural resource governance, especially in the face of high market demand and other drivers that have been shown to erode local governance arrangements (Cudney‐Bueno & Basurto 2009). Even in ideal contexts of collaborative management between the state and communities of resource users, conservation crime can be an intractable challenge for which natural resource governance disciplines have mostly failed to identify robust solutions. The application of scientific approaches to crime, like crime science, constitutes a promising avenue for innovative solutions to these persistent challenges, in particular if novel insights are developed in light of knowledge from natural resource governance disciplines that could both enhance the effectiveness of applied research and science–policy transfer and encourage critical reflexivity about the moral and ethical dimensions of those applications.

Crime science is one offshoot of criminology that includes the application of scientific methods, study of crime and security problems, and an aim of reducing harm—a concept incorporating loss or damage that can be physical, emotional, reputational, and social (Cockbain & Laycock 2017). Crime science has been successfully applied to reduce crime rates associated with homicide, interpersonal violence, and automobile theft. Crime science is consistent with more recent conceptualizations of crime that recognize rule of law violations as well as other forms of disorder, risk, and harm (e.g., Gibbs et al. 2010). One of the strengths of crime science is that it considers violations and harms to be nonrandom and to be a function of environmental factors that facilitate, promote, or provoke criminal events as well as individual criminal propensity (Cockbain & Laycock 2017). This perspective fits well with the context of conservation and conservation crimes, which typically are researched and managed according to specific spaces, such as biodiversity hotspots (e.g., Andes Mountains Tropical Hotspot) (e.g., Myers et al. 2000), or species at risk of extinction, such as the vaquita (*Phocoena sinus*) (e.g., D'agrosa et al. 2000). Thus, it is unsurprising that criminological and conservation sciences have been combined and are applied to on‐the‐ground conservation problems. Theoretically, such interdisciplinarity helps advance scientific understanding about crime and deviance and provides new avenues for discovery. Practically, such interdisciplinarity offers novel tools for diverse stakeholders working to address socioecological conservation problems. Moreto (2015) highlighted how “conservation crime science” could, for example, enhance novel integration of intelligence into decision‐making by park rangers in Uganda. In many ways, the integration of crime science, and associated scientists, into conservation can be viewed as a positive development. Diversity advances invention, the robustness of scientific findings, the enterprise of science, and the ability of scientists to produce socially relevant impacts (Cockbain & Laycock 2017).

## Breadth of Integrating Crime and Conservation Science

We used Google Scholar's advanced search option and its related articles feature to identify the breadth of peer‐reviewed literature integrating crime and conservation science for 3 contexts involved in deviant human–environment relationships: fish, plants and wildlife, and timber. We did not restrict our search by geopolitical boundary or publication date. In Google Scholar, we searched for the keywords “*crime science*,” “*wildlife*,” “*fish*,” and “*logging*.” Google Scholar search engine optimization algorithms were applied and the entire contents of peer‐reviewed journal articles were searched. We excluded articles for which the dominant method or theory was not crime or conservation science (e.g., social marketing, psychology) based on a review of the abstract, keywords, and journal aims and scope if needed. The goal of this search was to capture a range of interdisciplinarity applications on how crime science is applied to conservation. We recognized the potential to miss some studies or approaches, particularly those from criminology, that do not include practical applications or an aim of reducing harm but rather sought to usefully build knowledge about the etiology of crime (see Kurland et al. [2017] for a comprehensive review of the literature).

We identified 6 crime science approaches being used in the conservation contexts of illegal, unreported, and unregulated (IUU) fishing; wildlife and plant crime; and illegal logging. The first of these articles was published in 2012. The 6 approaches were choice‐structured properties, CRAVED or CAPTURED, crime scripting, distance decay, risky facilities, and situational crime prevention (Table 1). Briefly, choice‐structured properties (Cornish & Clarke 1987) describe the characteristics of offenses that structure an offenders’ choices about alternative courses of action. Characteristics of offenses are differentially attractive and can be manipulated to reduce crime. The CRAVED or CAPTURED approach is a model that guides the study of theft choices (Clarke 1999) and is an acronym for factors that make an object attractive to thieves (concealable, removable, valuable, enjoyable, disposable). Crime scripts are a step‐by‐step review of how a specific crime is committed and are used to identify the complete sequence of decisions and actions prior to, during, and after the crime and the links between them (Cornish 1994; Viollaz et al. 2021). Distance decay suggests spatial patterns of deviant and harmful activity, whereby most harms or crimes are committed nearer rather than farther away from a criminal's normal activity areas (Van Koppen & De Keijser 1997). Risky facilities are locations where far more crime and disorder occur than in other locations (Clarke & Eck 2007). Finally, situational crime prevention focuses on the modification of the parameters of opportunities and constraints (e.g., effort, risks) of specific crime situations (Clarke 1980).

**Table 1:** Six crime science approaches being applied in scientific investigations of conservation crime based on a February 2020 review of peer‐reviewed journal articles.*

	Type of conservation crime					
	illegal, unreported & unregulated (IUU) fishing		wildlife and plant crime		illegal logging	
Crime science approach	example insight	reference example	example insight	reference example	example insights	example reference
Choice structured properties	Better port infrastructure, high vehicle traffic, large amounts of fish imports and exports, and weak governance influence offloading of illegal catch.	Marteache et al. 2015	NA	how offenders commit illegal logging and what factors influence their decision‐making process at microlevel, such as access to open or logging roads	Marteache & Pires 2020	
Crime scripts	Describe process of illegally removing fish from the sea and subsequent processing before seafood enters market.	Petrossian & Pezella 2018	process before, during, and after wildlife are traded illegally by different types of sellers: casual, transient, opportunistic, hidden, and professional	Ayling2013; Leberatto 2017	Logging and mining companies may play third‐party role as local guardian and prevent illegal logging.	Ayling 2013
CRAVED or CAPTURED	Illegally caught fish species were more concealable (sold through ports of convenience), abundant, enjoyable (more often found in recipes), and disposable (highly commercial).	Petrossian et al. 2015, 2018	theft choices underlying live parrot poaching and attributes of parrots that make them hot products, such as chicks that are easily removed from nests	Pires & Clarke 2012; Pires & Petrossian 2016	NA	
Distance decay	Multiple factors influence target selection for fishing poachers in marine protected areas, particularly distance to offender target selection.	Weekers et al. 2019; Davis & Harasti 2020	techniques of geographic profiling of buffer zones and distance decay as applied to foraging patterns of bats	Le Comber et al. 2006	NA	
Risky facilities	Port traits, including degree of enforcement activity, facilitate vessel entry and offloading of illegal catch.	Weekers et al. 2019; Thiault et al. 2020	high‐risk routes for wildlife trafficking can trace particular source and destination locations at a country level.	Kurland & Pires 2017	NA	
Situational crime prevention	There is a relationship between local situational factors and illegal fishing in 53 countries, including proximity to ports of convenience and number of commercially significant species found within territorial waters. Presence of legally fishing vessels does not deter illegal fishing activity.	Petrossian 2015	situations from the environment that create enabling conditions for wildlife and plant poaching, such as lack of effective human–wildlife conflict mitigation programs	Kurland & Pires 2017; Lavorgna et al. 2018	Aspects of the built and natural environment influence illegal logging patterns in space and time.	Kurland et al. 2018

* Search engine Google Scholar, no date range restriction.

## Depth of Interdisciplinary Integration

Although it is important for decision making to apply the knowledge gained from interdisciplinary research, such as the studies identified in Table 1, it is also important to identify the boundaries of existing innovation. These spaces emerge when one identifies questions that the extant knowledge base does not yet answer. The case of sea cucumber trafficking in Mexico illustrates at least 5 observations about how the integration of crime and conservation sciences could advance the theory and application of conservation crime science.

## Context of Sea Cucumber Conservation in Yucatan, Mexico

We chose sea cucumber fishing in Yucatan, Mexico, as a case study to explore integrative approaches because it exemplifies situations in which the application of crime science and its synthesis with natural resource governance disciplines could have substantial impact. Sea cucumber poaching and trafficking is a persistent challenge in Latin America and around the world. The sea cucumber fishery in the Yucatan Peninsula is one of the most recent stocks targeted for harvest and sale in the global market, making it a particularly timely case (Bennett & Basurto 2018). We have shared extramurally applied research experience with the local communities. Coauthor Bennett resided in various Yucatán fishing communities as a participant observer–researcher for >1 year (in addition to multiple other approximately 4‐week research trips over 6 years). Bennett attended cooperative meetings, participated in fishing, and conducted more than 300 surveys and interviews with cooperative leaders, fishers, and private sector firms and fish buyers (Bennett 2017; Bennett & Basurto 2018). Together, we last visited the region during the first quarter of 2020, when we convened a series of workshops and meetings with fishing communities, government, and other stakeholders to discuss and explore the applicability of crime science to addressing sea cucumber crime. In the workshops, we introduced approaches from crime science and worked together with groups of fishing cooperative members from 5 different communities to gather information and design innovative community‐based efforts to curb sea cucumber poaching and trafficking. Although the results of this research will be described in a separate publication, our efforts led to our reflections presented here (Thus, this work did not involve human subjects.).

Illegal fishing and overexploitation more generally have typified many sea cucumber fisheries, some of which have followed a boom‐and‐bust fishing pattern (Purcell et al. 2013). Sea cucumber stocks are increasingly overexploited at a global scale, driven by high market demand. They are considered a culinary delicacy in Asian cuisine and command very high retail prices (Purcell et al. 2012; Fabinyi et al. 2017), sometimes up to US$500/kg (retail price) when dried (De Greef 2018). Due to their nature as slow‐moving, slow‐maturing, benthic resources, fishing pressure can rapidly degrade populations, which in turn negatively affects community livelihood and environmental security. There are few examples of successful sea cucumber management outside the developed world (Purcell et al. 2013), one indication of a need for innovative conservation solutions.

One of the most recent waves of intense fishing exploitation targets 2 commercially valuable species present off the coast of Yucatán, Mexico: *Isostichopus badionotus* and *Holothuria floridana* (locally referred to as pepino café and pepino lápiz, respectively). There are 14 species of sea cucumber in the region, 5 have commercial importance (*Astichopus multifidus*, *Isostichopus badionotus*, *Holothuria floridana*, *Holothuria mexicana*, and *Holothuria grisea*). *Isostichopus badionotus* and *Holothuria floridana* registered the highest landings by far due to their desirability in Asian markets (Gamboa‐Álvarez et al. 2020). Both species are listed by the International Union for Conservation of Nature as species of least conservation concern. Although neither is listed under the Convention on International Trade of Endangered Flora and Fauna, a proposal to list many other species of sea cucumbers under Appendix II was entered in 2019. Globally, the biggest threat to sea cucumbers is overfishing to supply international markets with luxury foods, mainly in China, Hong Kong, Taiwan, Singapore, Korea, and Malaysia (Purcell et al. 2012). Sea cucumbers have a high nutritional value because they are high in protein, low in fat, and rich in amino acids (Rodríguez Forero et al. 2013). Habitat degradation and loss also contribute to sea cucumber declines.

The commercial sea cucumber fishery in Yucatán was established in 2012 and already shows signs of overexploitation from illegal fishing (i.e., poaching and trafficking). Fishing operations occur farther from shore, divers work at greater depths, and many fishers experience markedly lower catch per effort.

The Mexican National Fisheries Commission, with input from the National Fishery Institute (INAPESCA), has established stringent regulations to manage the fishery, including a spatially explicit permitting system, vessel quotas, short open seasons (approximately 7–14 days), size limits, and required documentation for transport and sale. However, the presence of high market demand has undermined enforcement and fueled criminality in the sea cucumber fishery. Illegally fished sea cucumbers from Mexico have been involved in as many as half of the open wildlife investigations in the U.S. Fish and Wildlife Service's Southern California region. Their value drives a thriving black market in which the U.S. is often a transit location between Mexico and Asian markets. Illegal sea cumbers are concealed inside vehicles and then small quantities are carried by pedestrians across the United States–Mexico border that are then consolidated into larger shipments on the U.S. side and shipped to Asia (National Geographic 2018). The U.S. serves as the nexus of sea cucumber trafficking between Mexico and Asia. In 2017, the U.S. Department of Justice arraigned a Tucson firm in federal court related to the illegal trafficking, conspiracy, false labeling, and criminal forfeiture and importation contrary of US$17 million worth of Mexican sea cumbers (USDOJ 2017). Meanwhile, violence, drug addiction, and severe injury and death from diving‐related accidents have proliferated (Bennett & Basurto 2018). These conditions decrease the sustainability of the fishery and help drive illegal activity.

Even alongside high rates of sea cucumber poaching and trafficking, Mexico's long history of local‐level collective action in fisheries management (Bennett 2017) provides a foundation for well‐organized fishing cooperatives to lead efforts to combat sea cucumber poaching and trafficking (Bennett & Basurto 2018). A recent long‐term ethnographic study of the sea cucumber fishery in Yucatán revealed that fishing cooperatives and other community groups across the Yucatán coast have already demonstrated substantial will and capacity to combat sea cucumber poaching and trafficking (Bennett & Basurto 2018). For example, in Celestún, wives of cooperative members formed an informal patrol group, alerting authorities of the presence and location of illegal sea cucumber processing camps.

In Yucatán, Mexico, fishing cooperatives invested substantial time, labor, and resources to monitor and reduce sea cucumber poaching within their fishing concessions. However, a lack of clear communication lines with formal authorities left them powerless to enforce fishery regulations in the face of armed poachers willing to use violence. At least 3 specific factors have impeded the success of cooperatives’ efforts to prevent trafficking: disorganized and haphazard patrols, lack of effective lines of communication with authorities, and lack of coordinated efforts between communities (as poachers move along the coast) (Bennett & Basurto 2018).

The case of the sea cucumber fishery in Yucatán is not unique; it is one among an increasing multitude of natural resource contexts around the world plagued by crime (Collins 2019). The sea cucumber case illustrates that community‐based policing holds promise for reducing conservation crime problems. Local monitoring and enforcement can be highly effective and efficient because local stakeholders observe each other as an inevitable by‐product of carrying out their day‐to‐day livelihood activities (Ostrom 1990; Gibson et al. 2005; Chhatre & Agrawal 2008). However, where highly valuable fisheries resources are concerned, local‐level monitoring is often insufficient to deter outside poachers, and a lack of strong linkages with government authorities can lead to failure (Cudney‐Bueno & Basurto 2009).

The proliferation of literature on comanagement in the realm of natural resources governance has been relatively silent on the issue of conservation crime and criminality, a clear point where both roles of resource users themselves and government are necessary to ensure sustainability and socially beneficial outcomes (Nunan et al. 2018). Comanagement literature affirms that ideally local communities should participate more in enforcement (e.g., Jentoft et al. 1998). Although local participation certainly has the potential to decrease the transaction costs of enforcement, there is little guidance (theoretical or otherwise) about how local enforcement should interact with formal legal regimes, prosecution of crime, and the physical risks of enforcement, especially in high‐stakes natural resource contexts (but see some limited suggestions in Pinkerton [1994], and McCay et al. [2014]). And although crime science is increasingly providing insights for addressing conservation crime, the benefits of these insights could be accelerated through a more thorough cross‐fertilization with knowledge from the field of natural resource governance. Ultimately, deepening interdisciplinarity between crime science and the natural resource governance disciplines can enhance understanding of what sustainable natural resource governance might look like in the context of high market pressures and other powerful and persistent drivers of conservation crime. The field of natural resource governance is itself multidisciplinary, engaging theory from political science and policy studies (e.g., common‐pool resource theory, comanagement) as well as human geography and political ecology (e.g., Ostrom 1990; Robbins 2011; Pomeroy & Berkes 1997; Armitage et al. 2009).

## Case Study Observations and Integrative Opportunities

First, unlike many of the traditional types of crime on which the development of crime science has been based (i.e., theft, person‐to‐person violence), which typically affect an easily specifiable set of individuals (i.e., property owners or victims of violence), sea cucumber poaching and trafficking in Mexico involves a broad range of harms (i.e., loss, misfortune, violence, damage) and victims (i.e., death, injury). Illegal take of a single animal may not harm the total population of animals or any individual fisher's livelihood. However, once illegal fishing reaches a certain threshold and volume, sea cucumber stocks may be diminished, which would affect many human livelihoods. The impact of illegal fishing on an entire sea cucumber stock may be temporally remote and confounded by other environmental and human causal factors. In the case of the sea cucumber fishery, the most direct victims are likely fishers adjacent to fishing grounds where stocks are overexploited, but other stakeholders such as traders, wholesalers, retailers, the general public, and fishers who target other species that may be affected by sea cucumber decline may also be thought of as victims. Important questions remain about how crime science, which has excelled at prescribing solutions for specific, serious, and geographically concentrated types of crime, can account for often diffuse impacts associated with natural resource governance. Uncertainty arises about implications for effective prevention measures, appropriate investments in enforcement, and prosecution in natural resource governance contexts.

Deeper integration of conservation and crime science can help determine how one can scale the relative specificity of crime science to the more diffused context of harms and victims associated with conservation crime. Natural resources are often common‐pool resources, which are defined by the difficulty associated with excluding individuals from using them, on the one hand, and the potential for each individuals’ use to subtract from the amount of resource available to other users, on the other hand (Hardin 1968; Ostrom 1990). As a result, conservation crime often generates multiple harms. It is rare to consider singular human victims from conservation crimes to which a law enforcement authority or criminal justice professional will respond, and the degree of harm may be heterogenous across stakeholder groups (e.g., direct resource users, communities). (Rhinoceroses may be an exception because in some instances individual animals are monitored on a near daily basis.) In many instances, harms cannot be traced to single criminal act but rather the cumulative impact of conservation crime over time on a resource base. These same factors affect the crime dimensions of conservation by muting incentives to report and respond to crime in natural resource and conservation contexts. In some cases, individuals may not even have legal standing to pursue action in response to conservation crime even if they have perceived a harm. In conservation crime, there tends to be great distances between the target of the crime (i.e., natural resource) and the victims, further muddying traditional conceptions of the relationship between criminal acts, victims, and appropriate responses. Principles of distance decay may be further adapted or retrained to this conservation crime context. Political theorists and behavioral economic researchers theorize and empirically investigate the challenges that these distributed incentives create for governing natural resources, especially when they take the form of common‐pool resources, as well as some potential solutions to overcoming these challenges (e.g., Ostrom 1990; Bowles & Gintis 2006), insights which could inform the application of crime science to these contexts.

Second, illegal sea cucumber fishing and trade occurs across many different overlapping and sometimes contradictory scales. Often, illegal fishing is carried out in localized fishing grounds pertaining to one community by vessels that have traveled from communities farther down the coast and that will return to those communities to process and sell catch. Subsequently, illegal transport and trade crosses state, national, or other boundaries. The victims affected most directly by the crime are those living in communities proximate to illegal fishing areas, even though a substantial portion of related criminal activity (processing, illegal trade, and even export) occurs beyond the local level. At the same time, regulatory regimes and fisheries science are passed down from national and state levels and affect local‐level knowledge and incentives and capacities for compliance, monitoring, and enforcement.

Interdisciplinarity may spark answers to the question of where and when scalar mismatches emerge in conservation crime behavior and governance. In many conservation crime contexts, there may be mismatches between the spatial scale of the criminal activity and natural resource systems and the jurisdictional scale at which natural resource management and crime prevention regimes operate. Ecologically relevant scales may not functionally map onto scales relevant to governance and other social process or the scale of criminal activity itself. These scales combine, interact, and yield different implications for studying, responding to, and preventing problems. Natural resource crimes are often transnational, whereas governance and crime prevention authorities often do not extend beyond the jurisdiction of nation‐states. Meanwhile, the natural resource systems themselves, victims, and offenders may be more localized across the illicit supply chain. It may be possible to scale up risky facilities analysis from micro‐ to meso‐ or even macrosystems, depending on the geographic scope of the conservation problem.

Third, in the sea cucumber fishery, illegal fishing and fish trade activity occurs in the same places and often appears quite similar to legal activities. For example, permitted and nonpermitted fishers may be fishing in the same areas and authorities lacking specific training may be unable to visually recognize the difference. Boat owners who possess legal permits may simply exceed harvesting quotas or copy permits to use more vessels. Vessels permitted to harvest sea cucumber may also harvest other species for which they are not permitted during the same fishing trip, such as lobster (*Panulirus argus*), octopus (*Octopus maya*), and finfish (e.g., snapper [*Lutjanus campechanus*] and grouper [*Epinephelus morio*, *Mycteroperca bonaci*]). These examples demonstrate some of the nuances of natural resource crime, which crime science is not wholly able to account for.

Novel insights about the dynamic and interactive nature of legal and illegal networks may be best explicated by interdisciplinary science. Unlike many types of crime (e.g., assault, burglary) that are decidedly illegal, natural resource and conservation crimes resemble and often occur alongside legal uses of natural resources. Use of natural resources can be sustainable, legal, governed, and generate benefits for different stakeholders. Oftentimes, illicit natural resource supply chains exploit legal supply chains; they hide in plain sight as products move from producers in source countries to consumers in destination countries. Because legal supply chains theoretically generate economic and other benefits for society, there is an obvious desire to support their resiliency. However, illegal products may be laundered through legal supply chains (i.e., trade‐based money laundering) and be comingled with each other during transshipment. Empirical identification of illegal networks is challenging, and efforts to disrupt illegal supply chains ideally do not disrupt legal supply chains. Furthermore, illegality itself often exists on a spectrum from fully legal to fully illegal, with gray areas further complicating recognition, responses, and prevention of natural resources and conservation crime. Different stakeholders (e.g., local users, government officials) may not share the same conception of what constitutes illegal activities regardless of what the rule of law specifies. CRAVED can generate information about illegal product characteristics that might distinguish signals from noise in intermingled supply chains (i.e., supply chains with both legal and illegal commodities). Crime scripts also distinguish discrete links along supply chains that could aid recognition of illegal from legal activities.

Fourth, in many contexts around the world, natural resource laws and regulations have been established without the consultation of natural resource users, their traditional ecological knowledge, or a sufficient understanding of local human–environment relations. Formal regulations have been implemented on top of—and in some cases have undermined—existing informal or local‐level governance systems. In some cases, natural resource governance regimes established by states or driven by international nongovernmental organizations have resulted in the criminalization of existing or traditional resource uses to the detriment of resource‐dependent people (Robbins 2011). In the case of the sea cucumber fishery, local monitoring and enforcement activities have been crucial in the absence of sufficient formal law enforcement capacity to monitor the entire spatial and temporal extent of potential illegal fishing activities. However, in Yucatán, Mexico, community‐based monitoring and enforcement has become untenable as the tendency for illegal fishers to possess firearms has increased. Furthermore, the lack of law enforcement on some legal dimensions of the fishery (e.g., quota limits) has undermined shared understandings between government and some fishing stakeholders regarding how the legality of certain dimensions of the fishery is defined (Bennett & Basurto 2018).

The resultant question from this observation is how can decision makers more directly factor in communities and their authority to define and control crime? Natural resource management is often decentralized because federal or state agencies lack sufficient capacity to effectively govern and due to an emphasis from scholarly and policy communities on community‐based management and broader global political economic processes over the last 4 decades. Community‐based conservation and natural resource management are not a perfectly functioning governance system, but they can produce sustainable benefits for people and natural resources under the right conditions. But if community‐based conservation systems are increasingly asked to manage conservation crimes, then communities may be newly tasked with monitoring crime rates, responding to and preventing crime, or partnering with crime control authorities they previously have not engaged with. Although responsibilities for conservation governance may be devolved to communities, these communities are not always vested with the law enforcement authority needed for conservation crimes (nor is it necessarily desirable for communities to undertake the associated risks). In some instances, communities do not want to be deputized or otherwise engaged in managing conservation crime, but they are left with few alternatives by provincial or federal agencies. Situational crime prevention incorporates informal guardianship of places into crime‐preclusion activities and may complement community‐based conservation schemes. It may also help offer additional conceptualizations of what constitutes benefits for communities.

Fifth, in the sea cucumber fishery of Yucatán, Mexico, the Comisión Nacional de Pesca establishes fishing regulations in consultation with the INAPESCA. Generally, INAPESCA is responsible for publishing assessments of which fish populations are commercially exploitable through the National Fisheries Chart (Carta Nacional Pesquera). For the sea cucumber fishery, INAPESCA conducts transect surveys evaluating sea cucumber density, size, and abundance to define appropriate fishing seasons and allowable fishing quotas. In this case, therefore, natural sciences play a key role in defining the legality of multiple aspects of natural resource use, including when it is legal and illegal to harvest the resource and the volume of sea cucumber that can be legally harvested. This observation engenders the question of how barriers can be overcome that inhibit incorporating natural science into the rule of law? The legality and illegality of natural resource use requires natural science insight in addition to the traditionally incorporated normative and social dimensions—this additive dimension may not necessarily be present for traditional crime contexts (e.g., homicide, larceny). Beyond social control, conservation crimes require considering the biology and ecology of natural resources and their ecosystems. The information burden on natural scientists is high; natural scientists are asked to offer parameters about what constitutes legal versus illegal levels of use and evaluate the impacts of crime on resources. Beyond parameterizing sustainable levels of use, natural scientists ideally communicate scientific information in a way that is meaningful for the development and refinement of the rule of law. Many crime science approaches are data driven or include data fusion; quantitative natural science data in particular may fit well within existing crime science analyses, such as risky facilities or distance decay. Furthermore, the tendency for crime science to prioritize community collaboration in generating an information base that enables effective crime prevention likely makes it amenable to including local ecological knowledge in the definition and implementation of regulations, an approach emphasized in natural resource governance (Berkes 2009).

## Leveraging Interdisciplinarity to Support Conservation Policy Agendas

Conservation crime is a globally distributed societal problem; few if any ecological or social systems are immune to risks from the phenomenon. It is understandable that financial, personnel, and other resources are being allocated for preventing and responding to conservation crime. Historically, the conservation community has relied on solutions such as laws, regulatory schemes, social marketing, trade bans, or comanagement regimes; however, it appears discipline‐specific mechanisms have not sufficiently reduced harms from conservation crime. Full achievement of sustainable development goal indicators (e.g., SDG 16) and multilateral strategy objectives (e.g., African Strategy on Combating Illegal Exploitation and Illegal Trade in Wild Fauna and Flora in Africa) remain unmet.

Nurturing interdisciplinary crime and conservation science will offer new breadth of innovation to help advance risk management programs and other policy agendas (e.g., Moreto 2015). New insights can be brought to bear on, for example, harmful patterns of sea cumber trafficking. Illuminating, describing, and classifying trafficking processes can help direct limited law enforcement resources or nurture collaborative partnerships focused on high‐risk areas. New ways of thinking about the drivers underlying sea cucumber trafficking may introduce novel opportunities to collect and measure data. Insight about how the built environment and other dimensions of place shape opportunities for sea cucumber trafficking can inform targeted crime prevention strategies that engage local community members as guardians.

The case of sea cucumber trafficking in Mexico also illustrates the potential of leveraging more diverse scientific understanding with practical solutions to societal problems. Integrating crime and conservation science promotes reexamination of the historical and current power relationships between resource users, traditional governance systems, and formal authorities, such as the state (e.g., traditional vs. community policing; crime prevention vs. crime response; the roles of gender, race, and class in crime control). Newfound insight may help support stakeholder relationships as they are stressed and tested by sea cucumber trafficking risks (e.g., corruption, violence). Critically reflecting on the unique scalar dimensions and coordination of disparate management authorities may help build, for example, the capacity of decision makers to identify unsustainable decisions about sea cucumber stock management. Finally, local stakeholders may better anticipate and prepare to account for the role of natural sciences and accommodate the subtleties and variability of how crime is defined with respect to the changing status of natural resource stocks (what is classified as illegal may change rapidly as stock status changes) as well as inevitable scientific uncertainty and the potential for contradictions between government and local ecological knowledge.

Deeper integration of crime and conservation science offers exciting avenues for research as well as evidence‐based decision making (Moreto 2015) designed to fill gaps between articulated goals and progress on the ground, for example, in meeting the UN Sustainable Development Goal targets. Conservation crime science offers a focus that cross‐cuts and links a number of Sustainable Development Goals (Life on Land, Life on Water, Justice and Equity), livelihoods, and food security. In 2015, the Mexican Government's Fisheries Management Plan for sea cucumber envisioned a long‐term solution to the existing problems of poaching, trafficking, and general overexploitation that integrates scientific knowledge, the participation of resource users, and commitment of governmental authorities to enable adaptive, ecosystem‐based, and collaborative resource management. Clearly, the political will and foundations for effective monitoring and enforcement exist among local stakeholders and government. Cases such as the Yucatán sea cucumber fishery face persistent and daunting challenges, and traditional tools have been demonstrably ineffective, but these challenges are not insurmountable. Addressing these and similar issues around the world will require further innovation, to which we think that interdisciplinary engagements between crime science and natural resource governance fields can contribute productively. There has never been a more opportune time to deepen integration of crime and conservation science.
